# Identification of Key Non-coding RNAs and Transcription Factors in Calcific Aortic Valve Disease

**DOI:** 10.3389/fcvm.2022.826744

**Published:** 2022-06-29

**Authors:** Shuai Guo, Erli Zhang, Bin Zhang, Qingrong Liu, Zhen Meng, Ziang Li, Can Wang, Zhaoting Gong, Yongjian Wu

**Affiliations:** State Key Laboratory of Cardiovascular Disease, Fuwai Hospital, National Center for Cardiovascular Diseases, Chinese Academy of Medical Sciences and Peking Union Medical College, Beijing, China

**Keywords:** calcific aortic valve disease, non-coding RNA, transcription factor, epigenetics, bioinformatics

## Abstract

**Background:**

Calcific aortic valve disease (CAVD) is one of the most frequently occurring valvular heart diseases among the aging population. Currently, there is no known pharmacological treatment available to delay or reverse CAVD progression. The regulation of gene expression could contribute to the initiation, progression, and treatment of CAVD. Non-coding RNAs (ncRNAs) and transcription factors play essential regulatory roles in gene expression in CAVD; thus, further research is urgently needed.

**Materials and Methods:**

The gene-expression profiles of GSE51472 and GSE12644 were obtained from the Gene Expression Omnibus database, and differentially expressed genes (DEGs) were identified in each dataset. A protein-protein-interaction (PPI) network of DEGs was then constructed using the Search Tool for the Retrieval of Interacting Genes/Proteins database, and functional modules were analyzed with ClusterOne plugin in Cytoscape. Furthermore, Gene Ontology-functional annotation and Kyoto Encyclopedia of Genes and Genomes-pathway analysis were conducted for each functional module. Most crucially, ncRNAs and transcription factors acting on each functional module were separately identified using the RNAInter and TRRUST databases. The expression of predicted transcription factors and key genes was validated using GSE51472 and GSE12644. Furthermore, quantitative real-time PCR (qRT-PCR) experiments were performed to validate the differential expression of most promising candidates in human CAVD and control samples.

**Results:**

Among 552 DEGs, 383 were upregulated and 169 were downregulated. In the PPI network, 15 functional modules involving 182 genes and proteins were identified. After hypergeometric testing, 45 ncRNAs and 33 transcription factors were obtained. Among the predicted transcription factors, CIITA, HIF1A, JUN, POU2F2, and STAT6 were differentially expressed in both the training and validation sets. In addition, we found that key genes, namely, *CD2, CD86, CXCL8, FCGR3B, GZMB, ITGB2, LY86, MMP9, PPBP*, and *TYROBP* were also differentially expressed in both the training and validation sets. Among the most promising candidates, differential expressions of ETS1, JUN, NFKB1, RELA, SP1, STAT1, ANCR, and LOC101927497 were identified *via* qRT-PCR experiments.

**Conclusion:**

In this study, we identified functional modules with ncRNAs and transcription factors involved in CAVD pathogenesis. The current results suggest candidate molecules for further research on CAVD.

## Introduction

Calcific aortic valve disease (CAVD) is the leading cause of aortic stenosis affecting 2.8% of the population aged 75 years or older (1). Aortic valve sclerosis, which is the early phase of CAVD, affects >40% of people older than 75 years of age. Moreover, 1.8–1.9% of patients with aortic valve sclerosis progress to clinical aortic stenosis annually (2). The available demographic and epidemiological data suggest that the number of patients with CAVD who are >70 or 75 years of age will double or even triple in the next 50 years, especially in developed countries (3). Once CAVD is initiated, the progression is ineluctable, and the prognosis is poor when symptomatic severe aortic stenosis has occurred.

Initially, CAVD was thought to results from the wear and tear of valvular leaflets, as well as the passive accumulation of calcium deposition. However, more recent evidence indicates that CAVD arises through an active process that involves lipoprotein deposition, chronic inflammation, and active leaflet mineralization (4). In recent decades, no therapies have been demonstrated to significantly suspend or reverse CAVD progression except for surgical aortic valve replacement or transcatheter aortic valve replacement, although not all patients can tolerate these procedures (5). The limitations of aortic valve replacement include a high operative risk for senile patients, perioperative complications, anti-coagulation therapy, and reoperation due to deterioration of the bioprosthetic valve.

microRNAs (miRNAs/miRs) and long non-coding RNAs (lncRNAs) are distinct forms of non-coding RNAs (ncRNAs) that have potential diagnostic and therapeutic value for various diseases. microRNAs consist of ~22 nucleotides and can silence specific mRNA expression by binding to 3′ untranslated regions of target mRNAs and forming a ribonucleic acid-induced silencing complex (6). Previous research revealed that in *Homo sapiens*, over 1,000 miRNAs are transcribed that target more than 5,300 genes, equivalent to 30% of the entire set of expressed genes (7). The widespread target distribution in the gene set guarantees the potential diagnostic, treatment, and prognostic value of miRNAs for CAVD. LncRNAs are over 200 nucleotides long and do not encode proteins RNAs (8). Analysis of data in the NONCODE database (a comprehensive lncRNA database) revealed 56,018 lncRNA genes that are in humans, which equates to over twice the number of protein-coding genes (9). In addition, lncRNAs are capable of interacting with chromatin-modifying complexes, affecting the conformation of nuclear domains, or activating transcriptional enhancers. Moreover, some lncRNAs can interfere with the transcriptional machinery, help maintain the structure of nuclear speckles, or participate in post-transcriptional regulation (10). With such intricate attributes and insufficient knowledge of these functions, additional research concerning the potential roles of lncRNAs in CAVD is urgently needed.

Transcription factors (TFs) are proteins that bind to DNA in a sequence-specific manner and regulate transcription. TFs function as the first step in DNA decoding and direct the interpretation of the genome. Additionally, many TFs serve as major regulatory factors that control cell types, developmental patterns, and specific biological processes. Genes encoding TFs comprise approximately 8% of the human genome and are closely related to a large number of diseases and phenotypes. In recent literature, TFs like SRY-box transcription factor 9 (SOX9), Sp1 transcription factor (SP1), GATA binding protein 6 (GATA6), forkhead box O1 (FOXO1), and BTB domain and CNC homolog 1 (BACH1) have been studied to explore their roles in the occurrence and development of various cardiovascular diseases (11–17). However, the roles of TFs in CAVD have not yet been fully investigated.

Bioinformatics is an efficient and robust tool for investigating potential pathogenic and therapeutic targets for cardiovascular diseases. Thus, in this study, we aimed to identify functional modules in CAVD and key ncRNAs and TFs that regulate such modules *via* bioinformatics analysis. The results of this research provide candidate molecules for further research on CAVD pathogenesis and treatment.

## Materials and Methods

### Data Resources

Microarray datasets, including CAVD samples, were searched in the Gene Expression Omnibus (GEO) database (https://www.ncbi.nlm.nih.gov/geo/). The inclusion criteria were as follows: (1) samples were collected from human aortic valves; and (2) samples included CAVD and control aortic valves. Datasets with a small sample size or not based on human gene-expression profiles were excluded. After acquisition of the datasets, Affymetrix Human Genome U133 Plus 2.0 Array [HG-U133_Plus_2 (GPL570)], including GSE12644 and GSE51472 containing most of the samples, were included in the final analysis (18–21).

### Identifying Differentially Expressed Genes

The robust multiarray average algorithm in the affy package of R software (version 4.0.5; https://www.r-project.org/) was utilized for background correction, quantile normalization, perfect match correction, and summarization (22). After annotation for probes, DEGs between CAVD and normal aortic valves were subsequently identified using lmFit and eBayes functions in the limma package (23, 24). For genes corresponding to multiple probes, gene expression levels were defined as the average value obtained using multiple probes. *P*-values were adjusted using the Benjamini–Hochberg method. Genes with |log_2_(fold change)| value of >1 and an adjusted *P*-value of <0.05 were considered significantly differentially expressed in GSE51472. For GSE12644, genes with a |log2(fold change)| value of >0.5 and an adjusted *P*-value of <0.05 were considered significantly differentially expressed. The identified DEGs were further examined using GEO2R, an interactive web tool that enables users to compare samples in GEO series to identify DEGs under different conditions. DEGs in both datasets were visualized by generating volcano plots and heatmaps using the ggplot2 and pheatmap packages of R software, respectively (25, 26).

### Gene Ontology and Kyoto Encyclopedia of Genes and Genomes Pathway-Enrichment Analyses

To investigate GO biological processes, cellular components, molecular functions, and KEGG pathways associated with CAVD, GO and KEGG pathway-enrichment analyses were performed using the clusterProfiler package in R (27). *P*-values were adjusted using the Benjamini-Hochberg method. GO terms and KEGG pathways were considered significantly enriched if the adjusted *P*-value was <0.05.

### Gene Set Enrichment Analysis

In addition, to conventional enrichment analysis based on hypergeometric tests focused on DEGs, all genes detected in our analysis were used for GSEA, which is relatively more comprehensive and sensitive. In this study, GSEA was conducted using the clusterProfiler package in R. Terms with a |normalized enrichment score| value of ≥1 and an adjusted *P*-value of <0.05 were considered significant enriched.

### Protein-Protein-Interaction Network Construction and Functional Module Identification

A PPI network of DEGs was constructed using the Search Tool for the Retrieval of Interacting Genes/Proteins (STRING) database (version 11.0), which integrates all known PPIs and predicts PPIs using various robust methods (28). Proteins disconnected with other nodes and interactions with scores lower than medium confidence level were excluded from the PPI network. The ClusterOne plug-in for Cytoscape, which employs a greedy growth process, was utilized to identify different functional modules in the PPI network based on cohesiveness scores (29). Functional modules with sizes of less than five proteins or *P*-values of >0.05 were excluded.

### Identifying Key ncRNAs and TFs

Interactions between ncRNAs and their target genes were obtained from the RNAInter database (30). For TFs, interactions were obtained from the TRRUST v2 database (31). Interaction pairs with scores of >0.5 were retained for further analysis. Furthermore, ncRNAs and TFs with <2 interactions between functional modules were excluded. Finally, hypergeometric test was performed with R to validate the significance (*P*-value < 0.05) of interactions between regulators (ncRNAs and TFs) and functional modules. NcRNAs and TFs interactions were obtained from the RNAInter database. For interaction pairs between ncRNAs and TFs with scores of >0.3 were retained for further analysis.

### Validating Key Genes and TFs

The weight (W) value of each gene was calculated as follows:


W=log2|(fold change)|*−log10(P value) *degree

Genes with high W values were considered as key genes in the PPI network. Genes with the top 15 W values in GSE51472 were externally validated with the independent validation sets, GSE12644. Receiver operator characteristic (ROC) curves were visualized using the pROC package in R to evaluate their abilities to distinguish CAVD from normal samples (32). The expression levels of TFs obtained in our analysis were verified with the training and validation sets.

### Human Aortic Valve Samples and qRT-PCR Experiments

NcRNAs and TFs interacted with equal to or more than 3 functional modules were regarded as most promising candidates. Since the differential expression and molecular functions of the most promising candidate in miRNA (miR-126-3p) has been validated in previous studies concerning CAVD, qRT-PCR experiments in this study focus on lncRNAs and TFs.

Human aortic valve samples of 7 CAVD and 6 control patients were collected from Fuwai hospital. As for the CAVD samples, all aortic valves were obtained from patients with severe symptomatic aortic valve stenosis. The severity of calcific aortic valve stenosis was confirmed by Doppler echocardiography. Calcification of stenotic aortic valves was confirmed by post-explant examination. Control aortic valves were obtained from patients undergoing surgical replacement because of pure aortic valve regurgitation. All control aortic valves were smooth and pliable, without calcification, thickening, and morphological abnormalities at post-explant examination. The protocol was approved by the Institutional Ethical Review Board of Fuwai Hospital. Written informed consent was obtained before the collection of aortic valve samples. After excision, samples were placed in liquid nitrogen immediately and subsequently stored at −80°C for later use.

Total RNA was extracted from aortic valve tissues using Trizol^TM^ reagent (Invitrogen, Catalog#15596018). Subsequently, 500ng of total RNA was reverse transcribed into cDNA by using PrimeScript^TM^RT Master Mix (TaKaRa, Catalog#RR036A). Expression of ncRNAs and TFs interact with equal to or more than 3 functional modules were detected by qRT-PCR using PowerUp^TM^ SYBR^TM^ Green Master Mix (Applied Biosystems, Catalog#A25742). Relative expression levels were performed using 2^−ΔΔCt^ method, with GAPDH as reference gene. Sequences of primers were obtained from previous high-quality studies and provided in Supplementary Table S1. Statistically significant was determined by unpaired Student's *t*-test at *P*-value < 0.05 and statistical tendency at *P*-value between 0.05 and 0.15.

### Constructing a Prediction Model

Considering the vast number of DEGs and limited samples, the glmnet package of R was used to fit a least absolute shrinkage and selection operator (LASSO) regression model to narrow the number of candidate genes (33). The response type was set to binomial, and alpha was set to one. Cross-validation was used to confirm the optimal penalty parameter, lambda. Genes and their coefficients were selected according to minimum binominal deviance. Validation of the LASSO regression model was performed with the GSE12644 dataset, and ROC analysis was performed to assess the efficacy.

## Results

### Identification of DEGs

**Figure 1:**
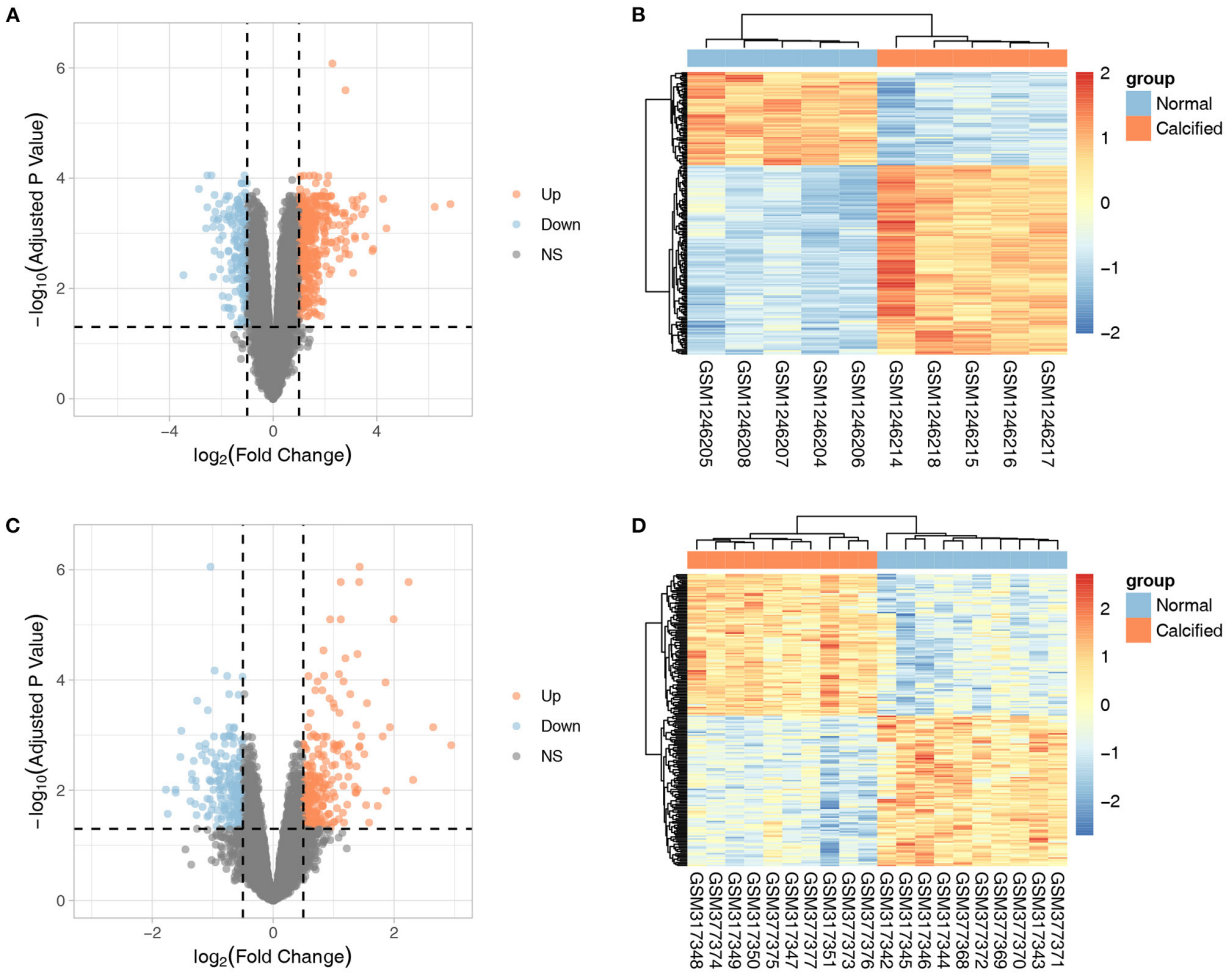
Volcano plots and cluster heatmaps of DEGs from the two datasets. **(A,B)** Volcano plots and cluster heatmaps of DEGs from the GSE51472 dataset. **(C,D)** Volcano plots and cluster heatmaps of DEGs from the GSE12644 dataset. In the volcano plots, the orange dots represent upregulated genes, and the blue dots represent downregulated genes. In the cluster heatmaps, the red bars indicate upregulated genes, and the blue bars indicate downregulated genes. The color gradation indicates the |log_2_(fold change)| value.

The flow-chart of this study was provided in Supplementary Figure S1. GSE51472 included 5 CAVD and normal samples each, whereas GSE12644 included 10 CAVD and normal samples each. Basic characteristics of both datasets were provided in the Supplementary Text S1. GSE51472 and GSE12644 were used to screen for DEGs after background correction, quantile normalization, perfect match correction, and summarization. No missing data were found for GSE51472 and GSE12644. In GSE51472, 552 DEGs were identified, of which 383 were upregulated and 169 were downregulated. In GSE12644, 429 DEGs were identified, including 245 upregulated and 184 downregulated genes. The DEGs of GSE51472 and GSE12644 were visualized by generating volcano plots, and the top 200 most significantly DEGs were shown in the cluster heatmap (Figure 1). Detailed expression profiles of the DEGs were shown in the Supplementary Table S2.

### GO and KEGG Pathway-Enrichment Analysis

**Figure 2:**
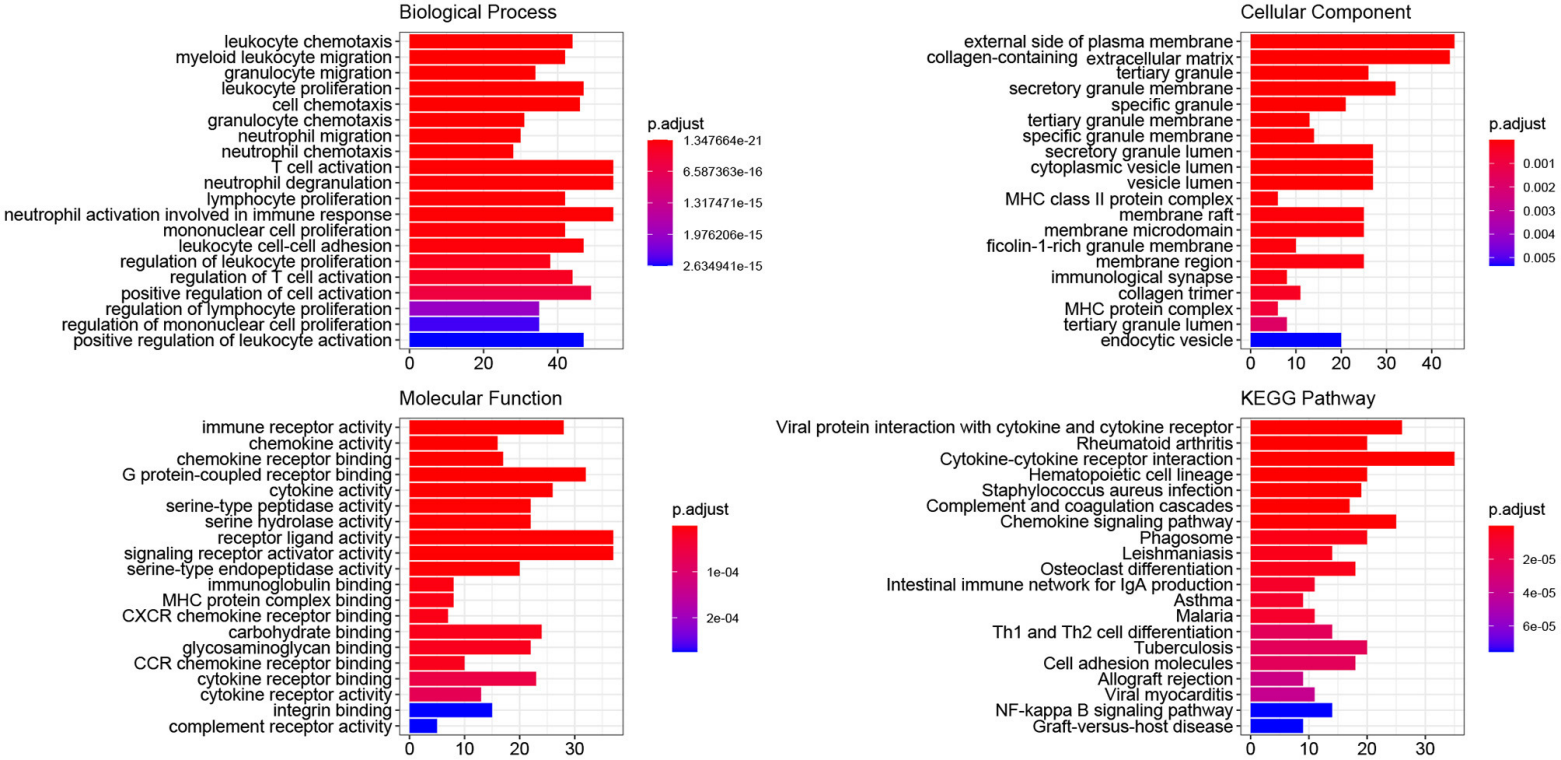
GO and KEGG pathway-enrichment analysis of DEGs. The colors, ranging from blue to red, represent adjusted *P*-value of each term, whereas the numbers in the X-axis indicate the number of DEGs for each specific term.

The ClusterProfiler package of R was utilized to conduct GO and KEGG pathway-enrichment analyses for the DEGs of GSE51472. GO terms included three major parts: biological processes (BPs), cellular components (CCs), and molecular functions (MFs). We found that 677 BP terms, 29 CC terms, and 69 MF terms, as well as 239 KEGG pathways were significantly enriched. Regarding BPs, the enriched terms mainly included immune response (GO:0030595, GO:0097529, GO:0097530, GO:0070661, GO:0060326), phagocytosis (GO:0006909), calcium ion homeostasis (GO:0055074, GO:0007204), calcium-mediated signaling (GO:0019722), and organization of extracellular matrix and structure (GO:0030198, GO:0043062). In terms of CCs, the top five enriched terms were external side of plasma membrane (GO:0009897), collagen-containing extracellular matrix (GO:0062023), tertiary granule (GO:0070820), granule membrane (GO:0030667), and specific granule (GO:0042581). With respect to MFs, the top five terms were immune receptor activity (GO:0140375), chemokine activity (GO:0008009), chemokine receptor binding (GO:0042379), G protein-coupled receptor binding (GO:0001664), and cytokine activity (GO:0005125). KEGG pathway-enrichment analysis indicated that CAVD shared pathways with rheumatoid arthritis (hsa05323), *Staphylococcus aureus* infection (hsa05150), leishmaniasis (hsa05140), and asthma (hsa05310). Furthermore, common vital signaling pathways such as the chemokine signaling pathway (hsa04062), NF-kappa B signaling pathway (hsa04064), IL-17 signaling pathway (hsa04657), Fc epsilon RI signaling pathway (hsa04664), Toll-like receptor signaling pathway (hsa04620), and T cell receptor signaling pathway (hsa04660) were also significantly enriched. Terms with the 20 smallest adjusted P-values in the BP, CC, MF, and KEGG pathways are shown in Figure 2, and the remaining terms are presented in the Supplementary Table S3.

### GSEA Findings

**Figure 3:**
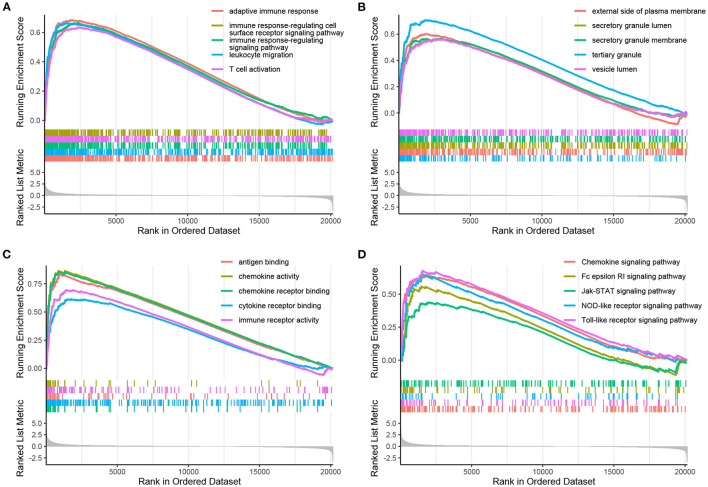
Enrichment plots obtained by GSEA. The enriched gene sets for BPs **(A)**, CCs **(B)**, MFs **(C)**, and KEGG pathways **(D)** are shown.

Using the filtering threshold mentioned above, 992 BP terms, 98 CC terms, 80 MF terms, and 53 KEGG pathways were identified. The top five BP, CC, and MF GO terms identified by GSEA are shown in Figures 3A–C, and the rest of the results are shown in the Supplementary Table S4. Regarding KEGG pathways, five common pathways are illustrated in Figure 3D, and other identified pathways are shown in the Supplementary Table S4.

### PPI Network Construction and Functional Module Identification

**Figure 4:**
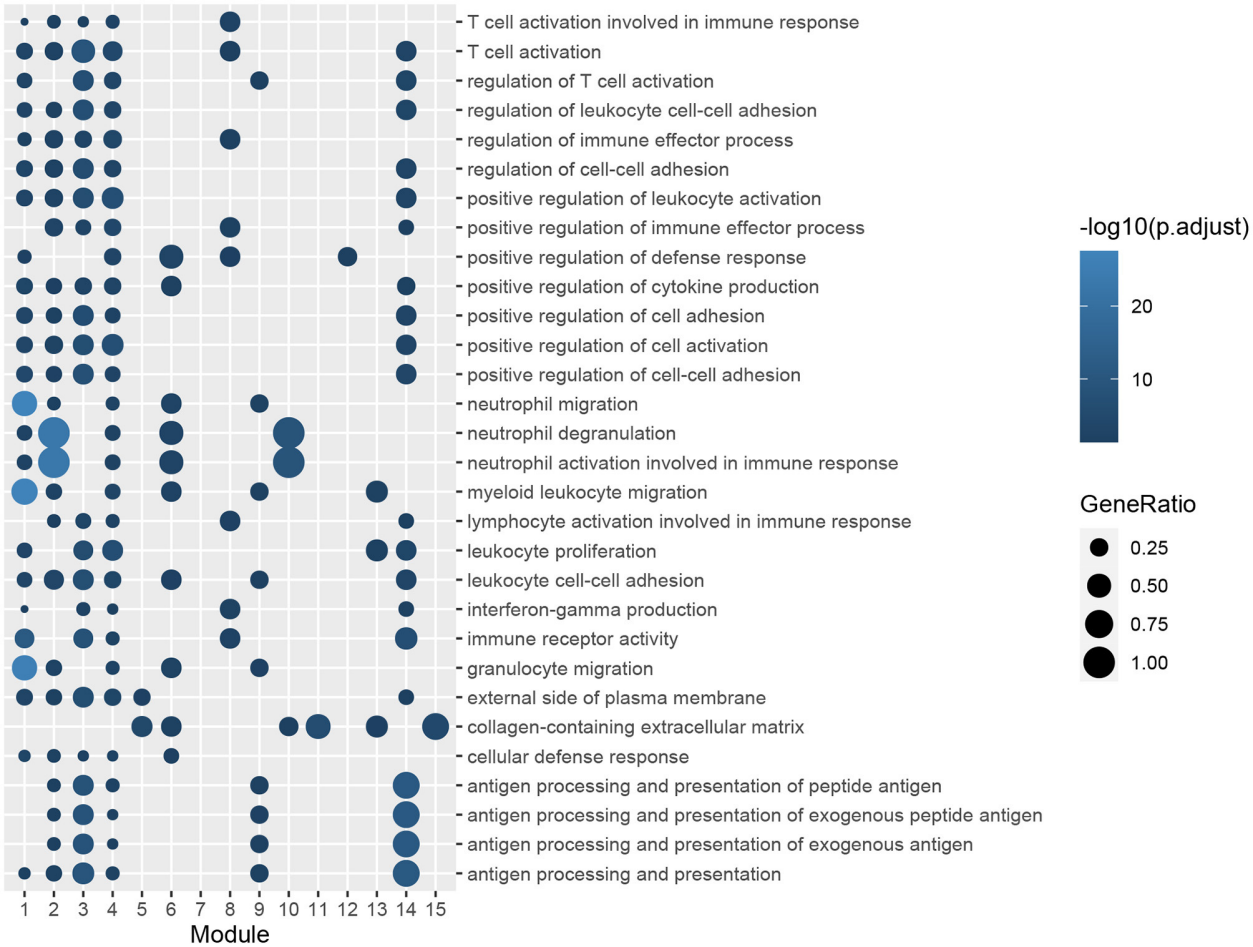
GO enrichment analysis of genes in functional modules. The color gradation represents the significance of the enrichment for each indicated GO term. The enrichment increased significantly, when going from a dark to a light shade. The sizes of the circles indicate the proportions of genes in each functional module present among the entered GO genes.

**Figure 5:**
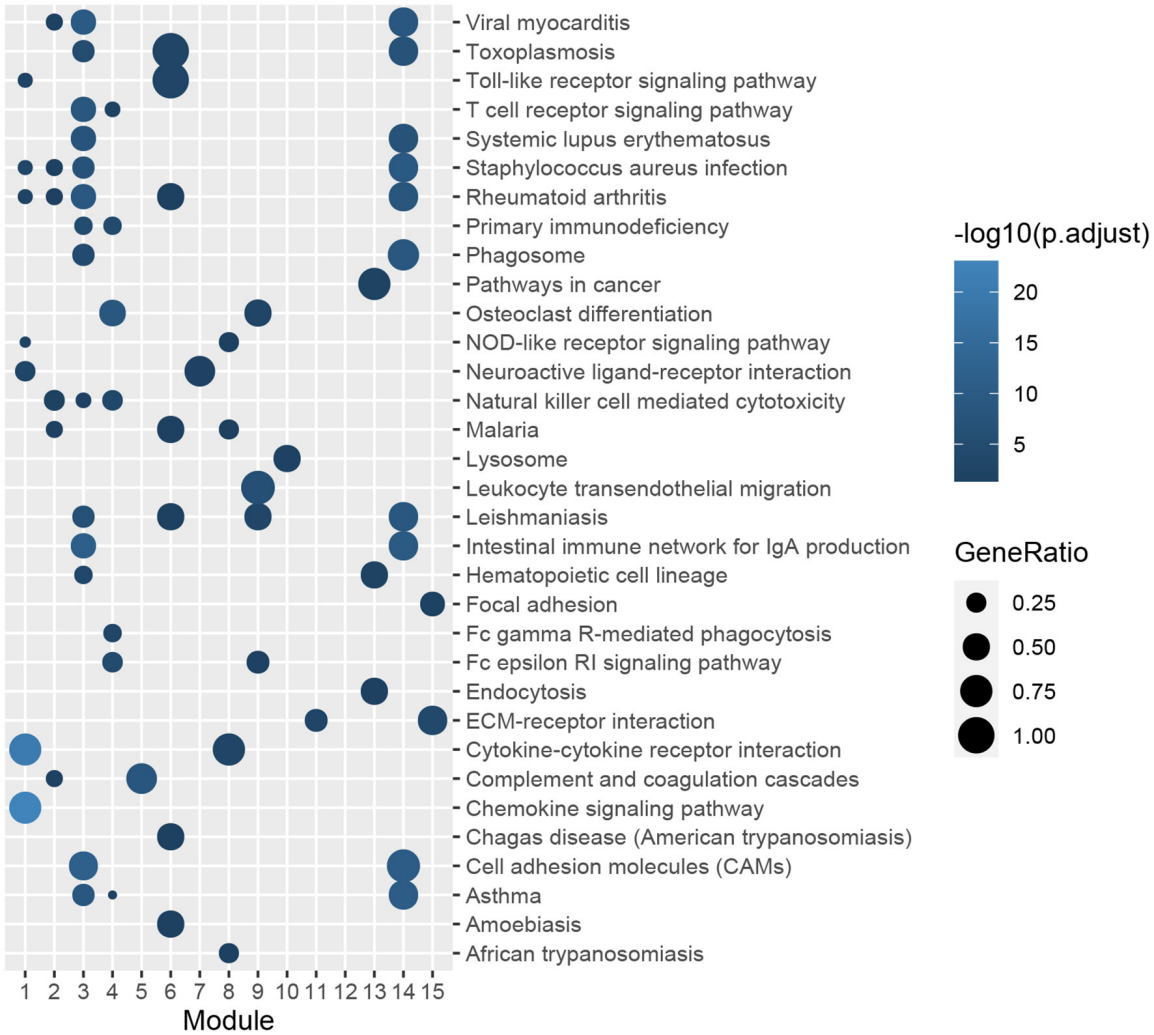
KEGG pathway-enrichment analysis of genes in functional modules. The color gradation represents the significance of the enrichment for each indicated KEGG pathway. The enrichment increased significantly, when going from a dark shade to a light shade. The sizes of the circles indicate the proportions of genes in each functional module present among the entered KEGG pathways.

A PPI network of DEGs in GSE51472 was established using the online STRING database with medium confidence. As mentioned above, proteins disconnected with other nodes and interactions with scores lower than the medium confidence level were excluded from the PPI network. Finally, 456 nodes and 4,472 edges were included in the PPI network. Then, we used the Cytoscape plug-in ClusterOne to identify the functional modules. Fifteen modules with sizes larger than or equal to five members and *P*-values of <0.05 were retained for further analysis (Supplementary Table S5). GO and KEGG pathway-enrichment analysis was performed for each module (Figures 4, 5). Furthermore, the 15 genes with the top W-values were considered as key genes in CAVD. These key genes included granzyme B (*GZMB*), *CD69*, matrix metallopeptidase 9 (*MMP9*), transmembrane immune signaling adaptor TYROBP (*TYROBP*), *CD86, CD8A*, Fc fragment of IgG receptor IIIb (*FCGR3B*), C-X-C motif chemokine ligand 13 (*CXCL13*), C-C motif chemokine ligand 4 (*CCL4*), lymphocyte antigen 86 (*LY86*), pro-platelet basic protein (*PPBP*), integrin subunit beta 2 (*ITGB2*), *CD2*, C-C motif chemokine receptor 7 (*CCR7*), and C-X-C motif chemokine ligand 8 (*CXCL8*) (Supplementary Table S6).

### Identification of Key ncRNAs and TFs

**Figure 6:**
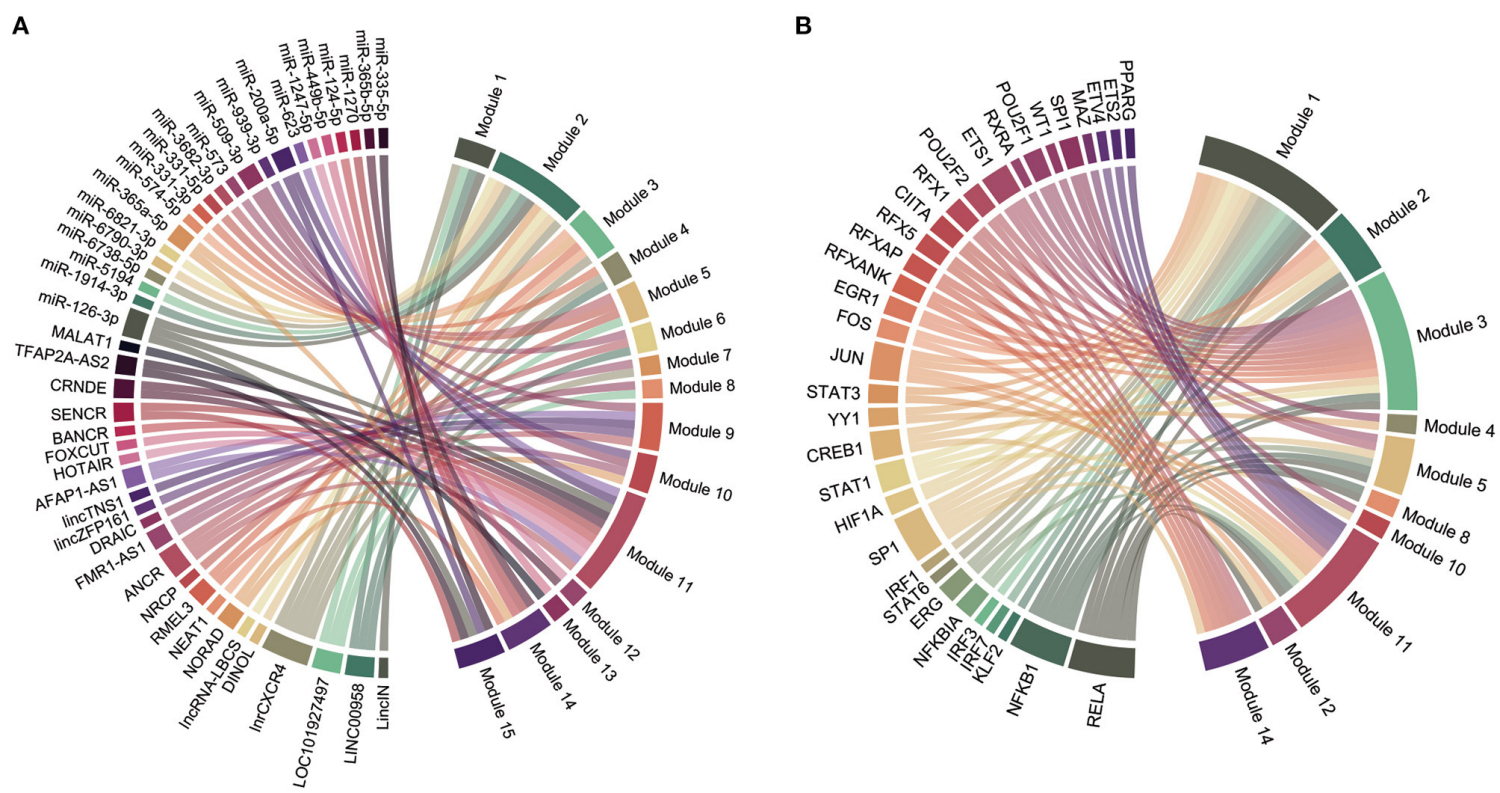
Regulatory chord diagram of key ncRNAs/TFs and functional modules. **(A)** Regulatory relationship between ncRNAs and functional modules. **(B)** Regulatory relationship between TFs and functional modules.

**Figure 7:**
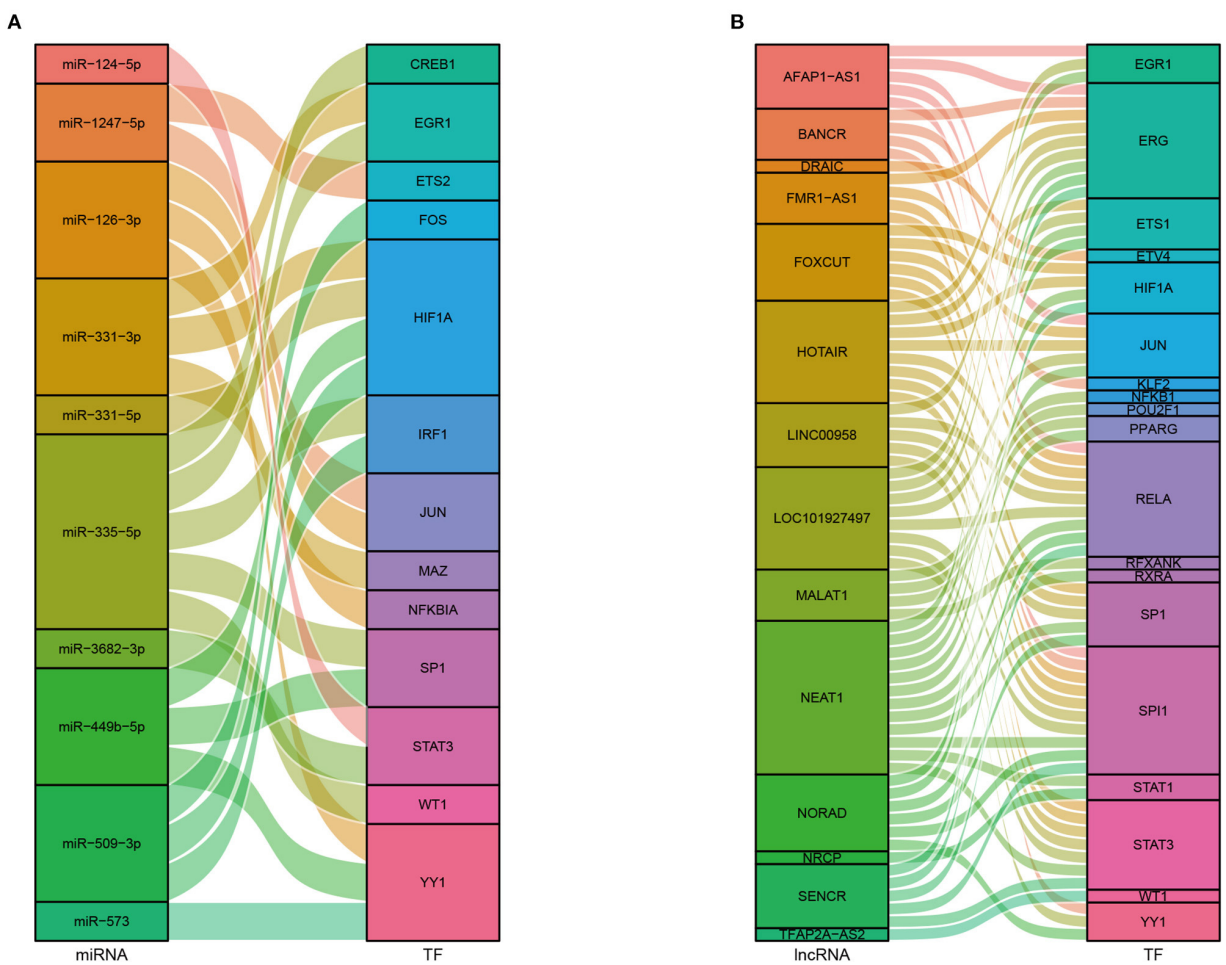
Sankey diagrams of the interactions between ncRNAs and TFs. **(A)** Interactions between miRNAs and TFs. **(B)** Interactions between lncRNAs and TFs.

We obtained 45 ncRNAs involving 67 ncRNA-module pairs after hypergeometric testing (Figure 6A). Notably, miR-126-3p interacted with three modules and miR-200a-5p, miR-509-3p, and miR-365a-5p interacted with two modules. Regarding lncRNAs, we found that lnrCXCR4 interacted with five modules, whereas ANCR, LINC00958, and LOC101927497 interacted with three modules. In addition, 33 TFs and 72 TF-module interactions were associated with the pathogenesis of CAVD (Figure 6B). Remarkably, RELA was found to interact with seven modules, and NFKB1, SP1, and JUN were found to interact with six, five, and four modules, respectively. Furthermore, a total of 93 ncRNA-TF interactions were identified (Figure 7). Among them, NEAT1 interacted with 12 TFs; both LOC101927497 and HOTAIR interacted with 8 TFs. For TFs, SPI1 interacted with 10 ncRNAs; RELA, ERG, and STAT3 interacted with 9 ncRNAs. To strengthen these interactions functionally, a network including ncRNAs, transcription factors, and functional modules was constructed (Supplementary Figure S2).

### Validation of Key Genes and TFs

**Figure 8:**
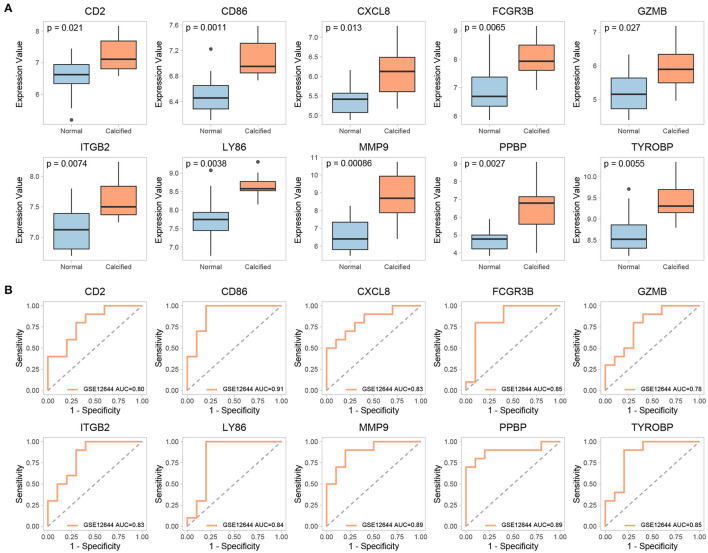
Validation of differential expression and ROC analysis of key genes. **(A)** Validation of differential expression of key genes in the GSE12644 dataset. **(B)** ROC analysis of common differentially expressed key genes in GSE12644 datasets.

**Figure 9:**
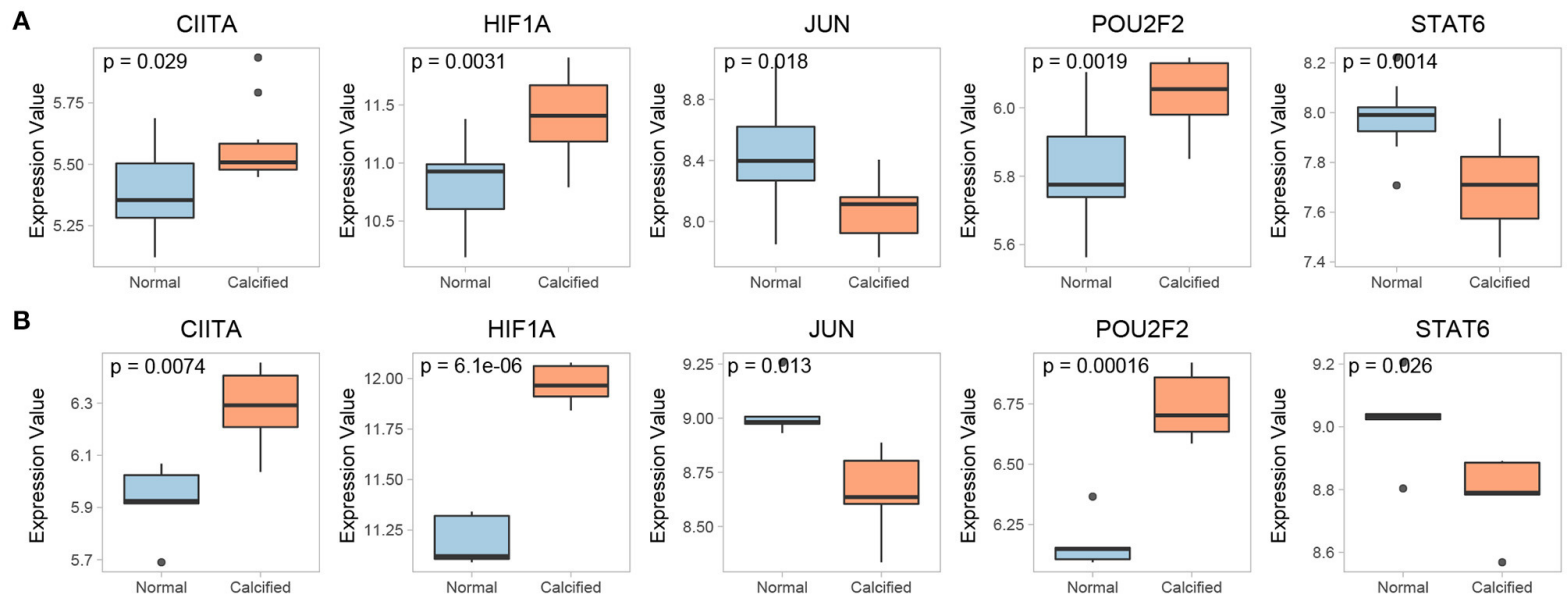
Commonly differentially expressed TFs in the **(A)** GSE51472 and **(B)** GSE12644 datasets.

The expression of 15 key genes in GSE51472 was externally validated using GSE12644. Among them, 10 genes were differentially expressed, which is consistent with the analysis of GSE51472 (Figure 8A). ROC curve analysis was performed to examine the potential of the gene to diagnose CAVD (Figure 8B). Among the TFs obtained by our analysis, CIITA, HIF1A, JUN, POU2F2, and STAT6 were significantly differentially expressed in both GSE51472 and GSE12644 (Figure 9).

### Validation the Expression of Most Promising ncRNAs and TFs Using qRT-PCR

**Figure 10:**
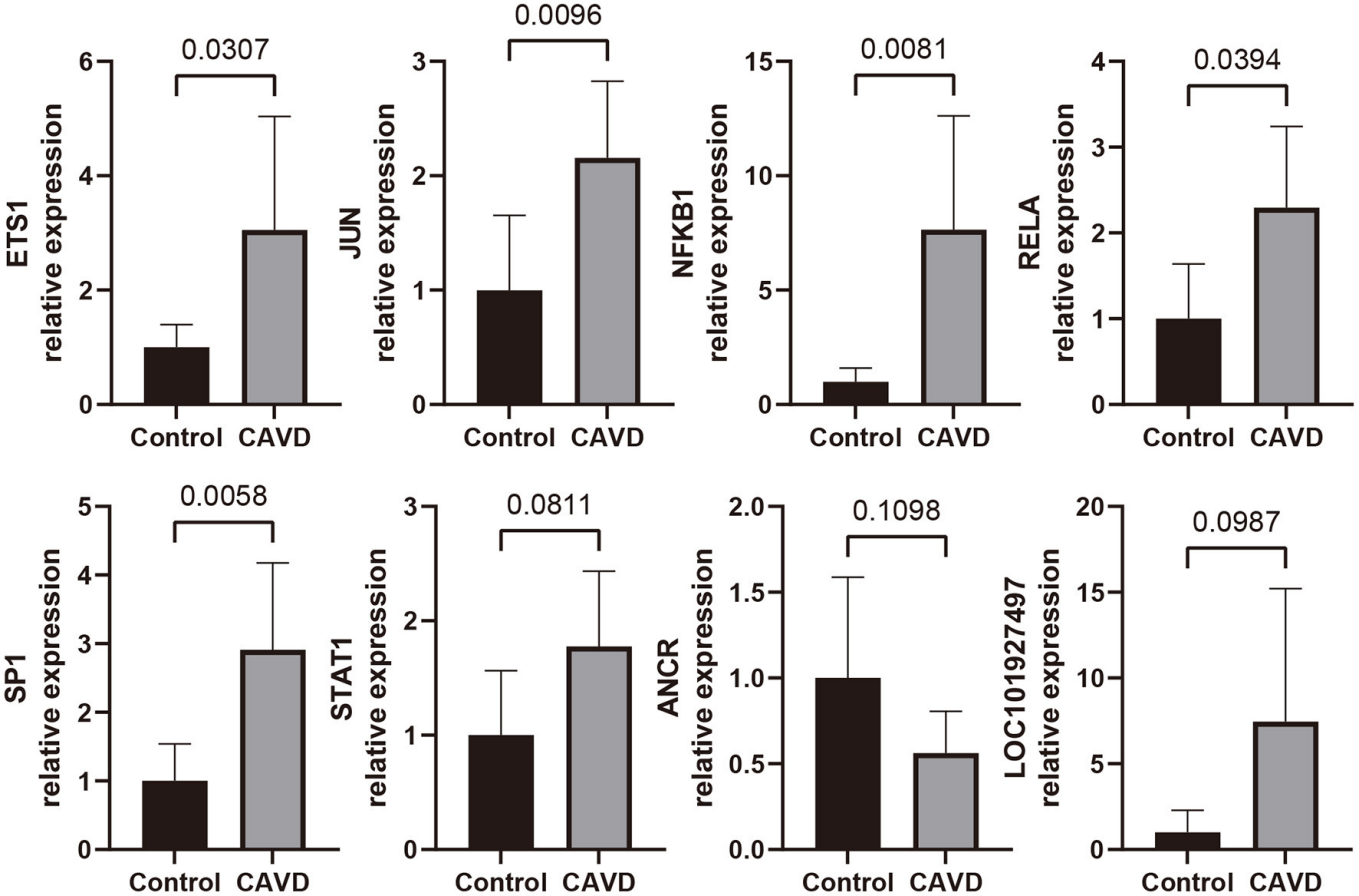
qRT-PCR analyses for expression level of the most promising candidate lncRNAs and TFs in control aortic valves (*n* = 6) and CAVD valves (*n* = 7).

To further validate the expressions of most promising candidate ncRNAs and TFs in CAVD and control valves, qRT-PCR experiments were performed. Relative expression levels of these candidates with statistically significance or statistical tendency were shown in Figure 10. The expression levels of ETS1, JUN, NFKB1, RELA, and SP1 in CAVD valves were significantly higher than those in control valves (*p* < 0.05). For STAT1, ANCR, and LOC101927497, statistical tendency was observed across two groups (0.05 < *p* < 0.15).

### Construction of a Prediction Model

**Figure 11:**
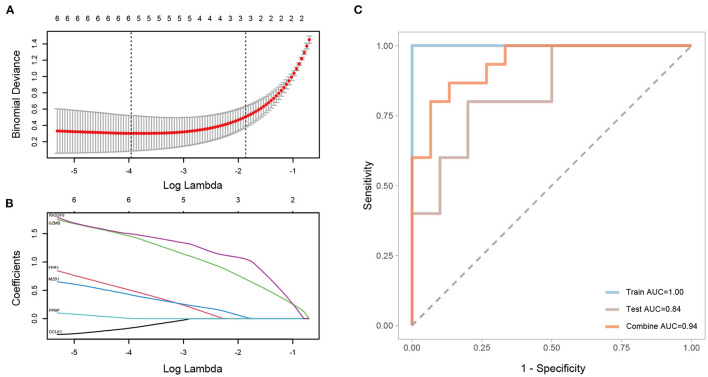
Construction and validation of a LASSO regression model. **(A,B)** Selected genes and their coefficients according to minimum binominal deviance. **(C)** ROC analysis of the LASSO regression model with the training set, validation set, and both sets combined.

A LASSO regression model was constructed for the DEGs identified between CAVD and normal samples in GSE51472, and the penalty coefficients were defined by the minimum binominal deviance. After penalty, doublecortin like kinase 1 (*DCLK1*), formyl peptide receptor 1 (*FPR1*), *GZMB*, marginal zone B and B1 cell specific protein (*MZB1*), pro-platelet basic protein (*PPBP*), and Ras association domain family member 6 (*RASSF6*) were retained to establish a diagnostic model with coefficients −0.1638865, 0.4981181, 1.4503176, 0.4198781, 0.0048172, and 1.4950435, respectively (Figures 11A,B). The area under the curve (AUC) of the diagnostic model was 1.00 in the training set, and that of the validation set was 0.84, and after combining the training and validation sets, the AUC was 0.94 (Figure 11C).

## Discussion

CAVD is characterized by ectopic mineralization and fibrogenesis of the aortic valve. Once CAVD deteriorates into severe aortic stenosis, the 5-year mortality rate reaches 67% (34). In addition to lipid-lowering using statins, potential therapeutic targets, including those targeting PCSK9/Lp(a), matrix gla-protein, soluble guanylyl cyclase, dipeptidyl peptidase-4, angiotensin II receptor, hydroxyapatite crystal formation, and RNAKL/osteoclastic activity, have been evaluated in randomized controlled trials for the treatment of CAVD. Although some studies observed attenuation of calcification burden, the effects on hemodynamic improvement require further studies (35). Certainly, the effectiveness of CAVD pharmacotherapy is still in its infancy, and surgical and transcatheter treatments also have limitations. Therefore, it is imperative to identify effective therapeutic targets for CAVD. With the capability to regulate gene expression, ncRNAs and TFs have a profound influence on the onset, development, and progression of CAVD. Thus, ncRNAs and TFs can potentially serve as therapeutic targets for CAVD.

Advances in transcriptomics are driving changes in preclinical and clinical medicine. The application of transcriptomics and the subsequent bioinformatic analysis of available data have identified precise molecular targets and annotated pathways, which revolutionized our understanding of initiation and progression of CAVD. In addition, transcriptomic studies provide mechanistic insights into valvular homeostasis as well as hypothetical basis for the diagnosis and treatment of CAVD, thereby guiding further research for this poorly understood disease (36, 37). As a widely used approach for transcriptomic analysis, microarrays have been used to discover pathogenic and therapeutic targets for cardiovascular diseases, including CAVD (38). In several previous studies, microarray-based gene-expression profiles were utilized to investigate hub genes, GO, and KEGG pathways in CAVD. However, further bioinformatic studies concerning ncRNAs and TFs in CAVD are needed. In this study, we not only identified key ncRNAs and TFs in CAVD, but also established a regulatory ncRNA/TF-target gene-pathway connection in CAVD for the first time.

In this study, the gene-expression profiles of CAVD and normal aortic valve samples were obtained from the GSE51472 and GSE12644 datasets in the GEO database. After data filtering, DEGs were identified using the lmFit and eBayes algorithms. In total, 552 DEGs in GSE51472 were identified, of which 383 were upregulated and 169 were downregulated. In GSE12644, 429 DEGs were identified, including 245 upregulated and 184 downregulated genes.

Subsequently, a PPI network of the DEGs in GSE51472 was established using the online STRING database. We identified 15 functional modules containing 182 members that were used for further analysis. Enrichment analysis demonstrated that the functional modules involved various GO terms and KEGG pathways, indicating the complex pathogenesis of CAVD.

We found that seven functional modules were involved in T cell regulation. It is well recognized that CAVD development occurs through a chronic immune-regulation process characterized by T lymphocyte infiltration and neovascularization. Compared with that in normal aortic valve tissues, greater activated-T cell infiltration was observed in calcified aortic valve tissues, and these T cells were densely distributed around calcified nodules (39). Similarly, recent data have shown that cytotoxic T cells can infiltrate into calcified regions and newly formed vasculature in calcified aortic valves, accompanied by expression of endothelial growth receptors and ossification (40).

Furthermore, six functional modules were related to the immune response of neutrophils. Neutrophils are the most abundant effector cells in the innate immune system and can secrete various pro-inflammatory factors that are harmful to tissues. Activated neutrophils can secrete free radical species and granules, which further leads to valve endothelial cell dysfunction and promotes immune cell infiltration into the valve (40). In addition, previous findings have indicated that the neutrophil: lymphocyte ratio in the peripheral blood is related to the degree of stenosis in the calcified aortic valve (41). Neutrophil extracellular traps (synthesized by neutrophils) were also observed in stenosed aortic valve tissues, accounting for 25% of all endothelial and subcutaneous cells in the tissues, and are also related to the degree of stenosis of the calcified aortic valve (42).

In addition, six modules involved collagen-containing extracellular matrix. Collagen disorganization in the extracellular matrix is a major hallmark of CAVD (43). Aortic valves are mainly composed of extracellular matrix and valvular interstitial cells. Three layers of connective tissue with different densities and molecular compositions form the basic structure of the aortic valve. The *fibrosa* is the outflow surface of the aortic valve and is mainly composed of type-I and type-III collagen fibers, which are the primary components responsible for valvular strength. The *ventricularis* is the inflow surface of the aortic valve and is mainly composed of elastin, which expands during diastole and rebounds during systole. The *spongiosa* is the core of the valve, and mainly contains loose connective tissue composed of glycosaminoglycans, which can absorb and buffer the relative shear movement of the *ventricularis* and *fibrosa* during periodic movement of the aortic valve (44). In CAVD, excessive deposition of collagen fibers mainly occurs in the *spongiosa*, and the number of collagen fibers can increase by over two-times, and the width and density of the fibers can also show corresponding increases. In contrast, previous data showed that in the *fibrosa*, the length of collagen fibers was obviously shorter, but that the number, width, density, or alignment did not change significantly (45).

Potential ncRNAs regulating each functional module were analyzed using a hypergeometric test, which identified 22 miRNAs and 23 lncRNAs. The downregulation of miR-126-3p, miR-335-5p, and miR-939 in calcified aortic valve tissue has been confirmed in previous studies (20, 46). However, the roles of these miRNAs in CAVD have not been fully explored. The present analysis not only identified that miRNA-126-3p plays an important regulatory role in CAVD but also that this role is mainly related to neutrophil degranulation, neutrophil activation involved in immune response, and the collagen-containing extracellular matrix. Consistent with these findings, Dimitry et al. reported that regulation of neutrophil function by miRNA-126-3p plays an important role in vascular homeostasis (47). Additionally, miRNA-126-3p can up regulate syndecan-4 in extracellular matrix, thereby exerting anti-atherosclerotic effects in vascular tissue (48). Furthermore, the present study showed that miR-335-5p is mainly involved in CAVD by affecting the collagen-containing extracellular matrix. This agrees with a previous study confirming that miR-335-5p inhibits NOTCH signal transduction by targeting JAG1 in atherosclerotic plaques, thereby reducing the degradation of collagen in extracellular matrix and stabilizing plaques (49).

Regarding lncRNAs, the lncRNA MALAT1 was originally studied in non-small cell lung cancer and was shown to be associated with tumor metastasis (50). Additionally, MALAT1 was found to be widely expressed in various organs and tissues. It has been confirmed that MALAT1 expression is upregulated in calcified aortic valves and human aortic valve interstitial cells after osteogenic induction. Mechanistically, MALAT1 upregulates SMAD family member 4 (SMAD4) expression by sponging miR-204, thus promoting the osteogenic differentiation of valve interstitial cells (51). However, in the present study, we found that the role of MALAT-1 in CAVD was associated with the collagen-containing extracellular matrix, which agrees with previous studies reporting the ability of MALAT-1 to regulate the extracellular matrix (52, 53). HOTAIR was the first lncRNA shown to act in trans to regulate gene expression (54). In recent years, many studies have suggested that HOTAIR is involved in the occurrence and development of cardiovascular diseases. HOTAIR is downregulated in human aortic valve interstitial cells exposed to cyclic stretch, and thus increases the expression of biomineralization associated alkaline phosphatase (ALPL) and bone morphogenetic protein 2 (BMP2), which leads to calcification of the aortic valve (55). Previous studies reported that HOTAIR regulates the collagen-containing extracellular matrix through URI1 activation of the Wnt pathway in myocardial fibrosis (56). Moreover, in thoracic aortic aneurysms, HOTAIR is closely associated with the expression of collagen types I and III in the extracellular matrix (57). Similarly, the present results indicated that HOTAIR mediates CAVD by acting on the collagen-containing extracellular matrix. AFAP1-AS1 was shown to be upregulated in calcified aortic valves and after osteogenic induction in human valvular interstitial cells (VICs). Overexpression or knockdown of AFAP1-AS1 can promote or inhibit the osteogenic differentiation of VICs, respectively. Mechanistically, AFAP1-AS1 upregulates SMAD family member 5 (SMAD5) expression by sponging miR-155, which eventually leads to the osteogenic differentiation of VICs (58). In addition, some data have also indicated that AFAP1-AS1 promotes the osteogenic differentiation of VICs by regulating macrophage polarization (59). Furthermore, the interaction between osteogenic and osteoclast differentiation is closely related to the calcification of vessels and valves (60). The present study indicated that in addition to common immune-related pathways and the collagen-containing extracellular matrix, AFAP1-AS1 is closely related to osteoclast differentiation in CAVD.

In this study, we identified 33 TFs that regulate functional modules associated with CAVD. Previous *in vitro* experiments indicated that valve endothelial cells on both sides of the aortic valve leaflet showed differential side-specific gene expression, including that of Kruppel-like factor 2 (KLF2), which is an “atheroprotective” and “vasoprotective” TF. Additionally, previous studies noted that KLF2 can inhibit the expression of inflammation-related genes and regulate calcification caused by proinflammatory stimuli (61). Consistent with this, the present findings suggested that KLF2 mainly regulates inflammatory cell migration, cytokine receptor interaction, and chemokine signaling pathways in CAVD. Hypoxia inducible factor 1 subunit alpha (HIF1A), predicted in our study, is highly expressed in calcified areas of stenotic valves and can lead to pathological remodeling of valve tissues by upregulating the expression and activity of MMP9-NGAL (62, 63). HIF1A is also related to the inflammation and endodontic-mesogenic transition of human aortic valve endodontic cells (64). The present findings also highlight the association of HIF1A with inflammatory responses in CAVD. Importantly, the upregulation of HIF1A was validated in both our training and validation sets (Figures 9A,B). Strikingly, upstream of HIF1A, signal transducer and activator of transcription 1 (STAT1) also plays a key role in CAVD, consistent with our analysis. Under normoxic conditions, the combination of lipopolysaccharides and interferon-γ can increase STAT1 expression, thereby upregulating HIF1A, followed by up-regulation of cell adhesion molecules (CAMs) and induction of calcification, which is also consistent with the present results (65). RELA, also known as the NF-κB p65 subunit, is an important part of the NF-κB signaling pathway. Previous research confirmed that RELA expression was upregulated in calcified aortic valves (66). *In vitro* experiments have also suggested that RELA positively regulates the mineralization and inflammatory responses of VICs (67, 68). Mechanistically, RELA phosphorylated at Ser536 is recruited to the promoter of *BMP2*, a key osteogenic marker, and initiates an osteogenic program (67). Moreover, RELA can also induce the expression of pro-inflammatory cytokines, such as TNF-α, IL-6, IL-1β and IL-8, which partially agrees with the findings reported in the present study (66). Early growth response 1 (EGR-1) has also been shown to be upregulated in human calcific stenosis aortic valves, as well as in VICs treated with osteogenic media (69, 70). STAT3 is a JAK3-related TF. Knocking down STAT3 in valvular interstitial cells inhibited expression of the osteoblastic markers, alkaline phosphatase (ALP) and RUNX family transcription factor 2 (RUNX2), and decreased matrix calcium deposition after pro-calcific stimulation (71). Moreover, p-STAT3 was also upregulated in calcified aortic valves and has clinical potential to inhibit the progression of valve calcification in an inflammatory milieu (66). Nuclear factor kappa B subunit 1 (NFKB1), an inflammation-related TF, was shown to be upregulated in aortic valve endothelial cells exposed to shear stress (72).

Fifteen genes with the highest *W values* in the GSE51472 dataset were verified using the GSE12644 dataset. Among them, 10 genes were differentially expressed in both datasets. Previous findings showed that abnormal expression of these genes was closely related to the occurrence and development of CAVD. CD86 is required for T cell activation by monocytes, and increased CD86 expression was accompanied by reduced osteoclast resorptive function and may contribute to calcification in CAVD (73). CXCL8, also known as IL-8, is a chemokine that facilitates the directed migration of cells to inflammatory sites (74). In addition to our observations that CXCL8 was upregulated in GSE51472 and GSE12644, data from a study also demonstrated higher CXCL8 protein expression in calcified aortic valve tissues than in normal tissues (75). Granzyme is a potent toxin expressed by cytotoxic lymphocytes, which can induce specific cell death-signaling pathways (i.e., activation of caspase-driven cell-death pathways). Among all granzymes, granzyme B (GZMB) is the fastest-acting and most effective pro-apoptotic granzyme (76). Previous qRT-PCR and immunohistochemistry data confirmed that GZMB expression was higher in calcified aortic valve tissues than in control valves (77). MMP-9 expression was increased in calcified stenotic aortic valves, which are mainly synthesized by tissue macrophages, suggesting an inflammatory state (78). MMP-9 is also present at significantly higher levels in the serum of patients with CAVD (79). Data from an *ex vivo* study confirmed that elevated cyclic stretching and fluid shear stress also stimulated MMP-9 expression in valve leaflets (80, 81).

The LASSO regression model was constructed and ROC curves were drawn for the training, validation, and combined sets, and the AUC values were all larger than 0.8, indicating the superior capability of using six DEGs (namely, *DCLK1, FPR1, GZMB, MZB1, PPBP*, and *RASSF6*) as biomarkers for distinguishing CAVD patients.

This study has some limitations. First, to strengthen the reliability of the results, we used one dataset as the training set and the other dataset as the validation set. However, the sample size was still small. Second, previous studies have identified stage-specific proteomic and transcriptomic signatures in CAVD, of which the calcific stage different from the non-diseased stage, and the fibrotic stage represents an intermediate state. Pathways enriched in calcific stage in CAVD were mainly associated with matrix metalloproteinase activation and MAPK signaling pathways. However, in fibrosis stage, pathways enriched were mainly associated with myofibrogenesis and oxidative stress of VIC. Given the existence of different pathogenic stages in a single CAVD leaflet, gene-expression data obtained using bulk transcriptomic profile may mask some real differences (36). Third, in addition to the pathological stage-specificity, the anatomic aortic valve microlayers (fibrosa, spongiosa, and ventricularis) also have unique proteomic features. These proteomic features were conserved between the CAVD and control valves but distinct between each individual layers. From the microscopic view of aortic valve, VICs in the fibrosa layer have stronger calcification potential compared to other anatomic layers, which can be explained by unique proteomic features (36). Furthermore, studies have also confirmed the layer-dependent sensitivity of VICs to dynamic strain, which may also suggest difference in gene expression between anatomical layers (82). Due to the inherent limitations of bulk transcriptomic profiling, this study was unable to provide more insight into the above-mentioned heterogeneity. Further bioinformatics analysis based on single-cell transcriptome data of CAVD may promote the progression of relevant research. Thus, gene-expression data from bulk transcriptomic profile may cover some of the true differences. Finally, the original data lack detailed clinical information, which may shed new light on future research when combined with the present study.

In conclusion, many results of the present study are consistent with those reported previously, confirming the reliability of our analysis. Furthermore, our study provides novel insight into molecules confirmed as playing important functions in CAVD. More importantly, we included ncRNAs and TFs in the present analysis that had not previously been evaluated in CAVD, thereby providing candidate molecules for further study.

## Data Availability Statement

Publicly available datasets were analyzed in this study. This data can be found here: https://www.ncbi.nlm.nih.gov/geo/query/acc.cgi?acc=GSE51472; https://www.ncbi.nlm.nih.gov/geo/query/acc.cgi?acc=GSE12644.

## Ethics Statement

The studies involving human participants were reviewed and approved by Ethics Committee of Fuwai Hospital. The patients/participants provided their written informed consent to participate in this study.

## Author Contributions

SG, EZ, BZ, and QL designed this study. SG, ZM, ZL, CW, and ZG contributed to the data analysis and manuscript preparation of this study. YW is an expert in the field of valvular heart disease who offered experience and knowledge for this study. All authors have reviewed the submitted manuscript, contributed to the article, and approved the version.

## Funding

This study was supported by grants from the National Natural Science Foundation of China (NSFC) (82170378) to EZ, from NSFC (81970326) to YW.

## Conflict of Interest

The authors declare that the research was conducted in the absence of any commercial or financial relationships that could be construed as a potential conflict of interest.

## Publisher's Note

All claims expressed in this article are solely those of the authors and do not necessarily represent those of their affiliated organizations, or those of the publisher, the editors and the reviewers. Any product that may be evaluated in this article, or claim that may be made by its manufacturer, is not guaranteed or endorsed by the publisher.

## Abbreviations

CAVD: calcific aortic valve disease
DEGs: differentially expressed genes
GEO: Gene Expression Omnibus
GO: Gene Ontology
GSEA: Gene Set Enrichment Analysis
KEGG: Kyoto Encyclopedia of Genes and Genomes
LASSO: least absolute shrinkage and selection operator
lncRNA: long non-coding RNA
miRNA: microRNA
ncRNA: non-coding RNA
PPI: Protein-Protein-Interaction
qRT-PCR: quantitative real-time polymerase chain reaction
STRING: Search Tool for the Retrieval of Interacting Genes/Proteins
TF: transcription factor
VIC: valvular interstitial cell.
