# Supplementary figures and images for: Identification of Key Non-coding RNAs and Transcription Factors in Calcific Aortic Valve Disease
Source: Front Cardiovasc Med. 2022 Jun 29;9:826744. doi: 10.3389/fcvm.2022.826744 (PMC9276990; doi:10.3389/fcvm.2022.826744)

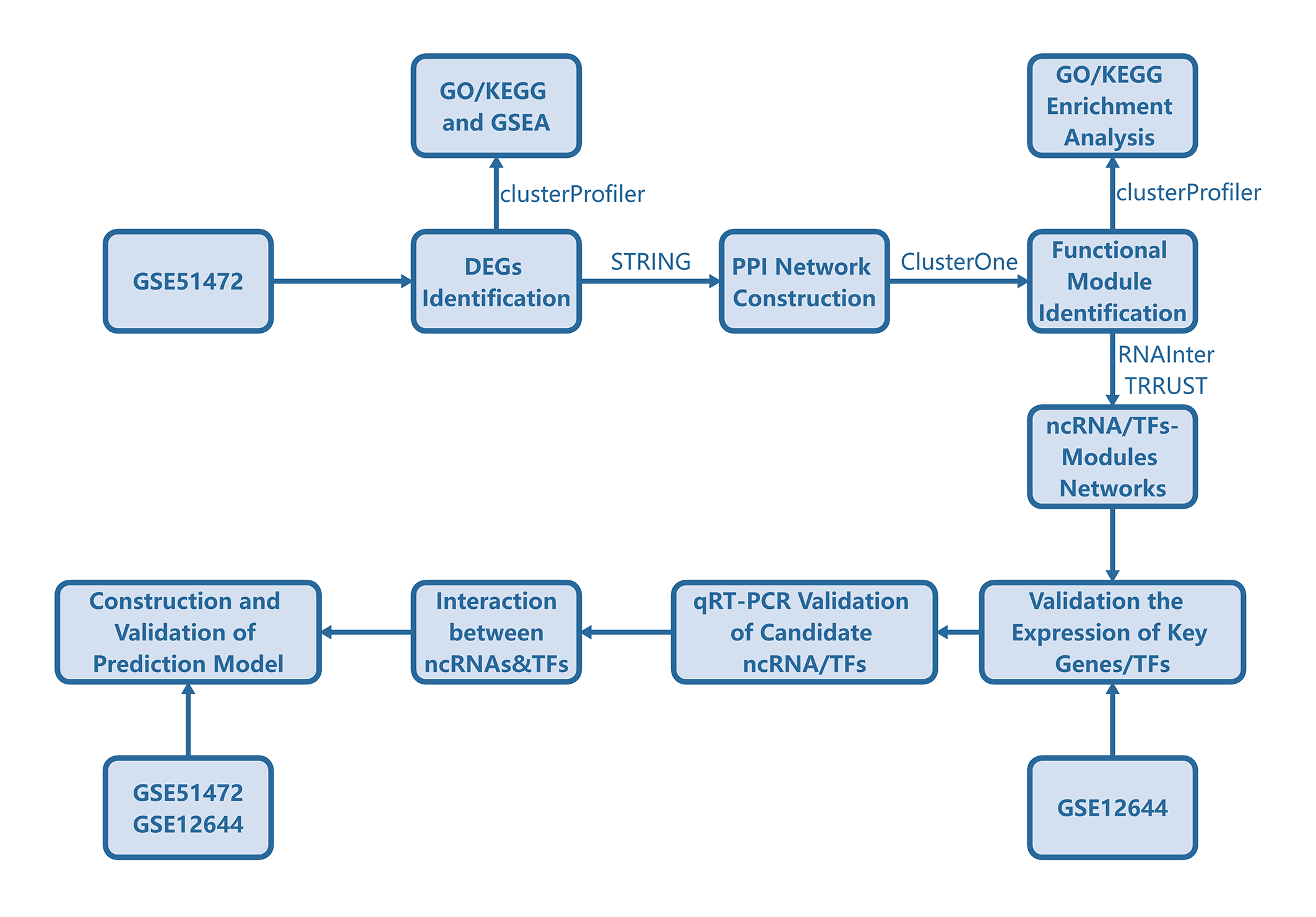

Supplement: Supplementary file 1 [file Image_1.TIF]

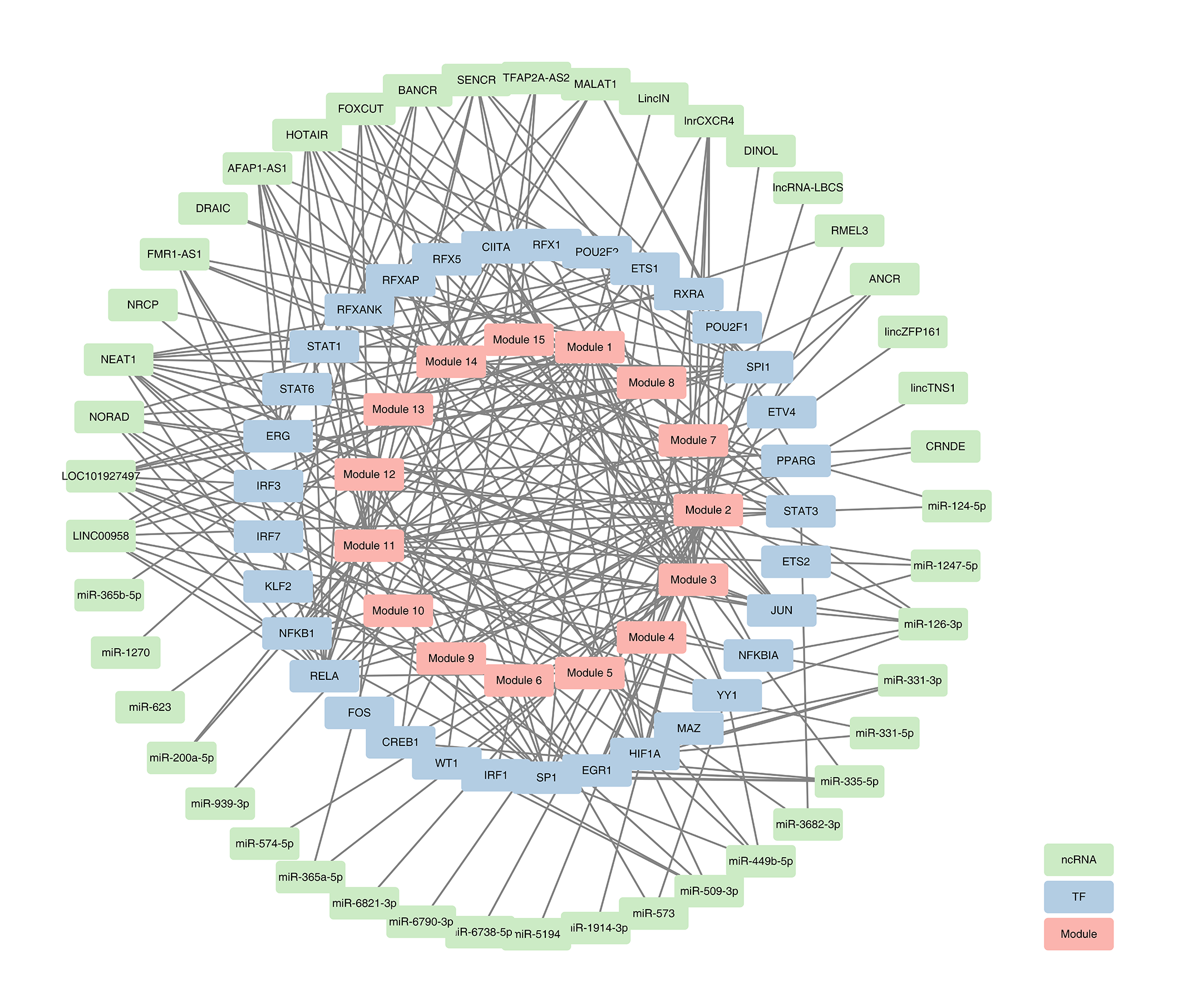

Supplement: Supplementary file 2 [file Image_2.TIF]
