# Supplementary material for: Identification of Key Non-coding RNAs and Transcription Factors in Calcific Aortic Valve Disease
Source: Front Cardiovasc Med. 2022 Jun 29;9:826744. doi: 10.3389/fcvm.2022.826744 (PMC9276990; doi:10.3389/fcvm.2022.826744)
Supplement: Supplementary file 9 [file Table_7.DOCX]

Characteristics for GSE51472 and GSE12644

|  | GSE51472 | GSE12644 |
| --- | --- | --- |
| Tissue | Calcific/control aortic valves | Calcific/control aortic valves |
| Species | *Homo sapiens* | *Homo sapiens* |
| Sample size | 5 + 5 | 10 + 10 |
| Country | Finland | Canada |
| Contributors | Rysä J | Bosse Y, Pibarot P, Mathieu P |
| Methods | Expression profiling by array | Expression profiling by array |
| Platforms | Affymetrix Human Genome U133 Plus 2.0 Array | Affymetrix Human Genome U133 Plus 2.0 Array |

Patient characteristics for GSE51472

|  | Non-calcified Control | Calcified Aortic Valves |
| --- | --- | --- |
| No. of patients | 5 | 5 |
| Male | 5 (100%) | 5 (100%) |
| Age (yrs) | 43.8±18.4 (31-69) | 56.8±9.9 (45-72) |
| LVEF (%) | 56.6±6.5 | 57.8±14.9 |
| DM | 0 | 0 |
| Coronary disease | 0 | 1 (20%) |
| ASO | 0 | 0 |
| COPD | 0 | 0 |
| Statins | 0 | 0 |

Values are mean ± standard deviation (SD) for continuous variables, number (percentage) for categorical variables. LVEF, left ventricular ejection fraction; DM, diabetes mellitus; ASO, peripheral atherosclerosis; COPD, chronic obstructive pulmonary disease.

Patient characteristics for GSE12644

|  | Non-calcified Control | Calcified Aortic Valves |
| --- | --- | --- |
| No. of patients | 10 | 10 |
| Male | 10 (100%) | 10 (100%) |
| Age (yrs) | 58.6±11.1 | 62.5±8.6 |
| BMI (kg/m^2^) | 24.9±4.2 | 25.9±4.1 |

The workflow of sample processing

For GSE51472, aortic valves were derived from patients undergoing aortic valve or aortic root surgery. After removal, aortic valves were quickly placed in liquid nitrogen and subsequently stored at -70°C for later use. Total RNA from valve tissue was extracted using the guanidine thiocyanate-CsCl method, followed by purification using the RNeasy Mini Kit (Qiagen) according to manufacturer's instructions. The quality and integrity of total RNA were analyzed by gel electrophoresis (Genom Data. 2015 Dec 19;7:107-8. doi: 10.1016/j.gdata.2015.12.015.).

For GSE12644, calcified valves were derived from patients with severe aortic stenosis undergoing valve replacement, and normal noncalcified aortic valves were derived from heart transplantation or aortic replacement. All valves were stored in the local biobank at -80°C. RNA was extracted from 100 mg of valve tissue using the RNeasy Lipid Tissue Kit (QIAGEN, Mississauga, Ontario) according to the instructions of the operating manual. Subsequently, the quality of extracted RNA was assessed using the Agilent 2100 Bioanalyzer (Circ Cardiovasc Genet. 2009 Oct;2(5):489-98. doi: 10.1161/CIRCGENETICS.108.820795.).
